# Over-the-wire technique in ERCP for common bile duct stone after total gastrectomy with jejunal interposition

**DOI:** 10.1055/a-2058-8809

**Published:** 2023-04-26

**Authors:** Yuki Ikeda, Toshinori Okuda, Norito Suzuki, Ginji Oomori, Shota Yamada, Shinya Minami

**Affiliations:** Department of Gastroenterology, Oji General Hospital, Tomakomai, Hokkaido, Japan

The main reconstruction procedure presently used after total gastrectomy is the Roux-en-Y method, but jejunal interposition was previously performed 1 . The most appropriate technique for endoscopic retrograde cholangiopancreatography (ERCP) after jejunal interposition has not been determined because of a lack of reported cases.

A 70-year-old woman with a history of jejunal interposition after total gastrectomy was hospitalized for a common bile duct stone. Although ERCP was attempted using a short-type double-balloon endoscope (EI-580T; Fujifilm Holdings Corp, Tokyo, Japan), biliary cannulation failed despite reaching the duodenum. Laparoscopic exploration of the common bile duct and cholecystectomy were subsequently performed. After 2 years, the patient presented with a recurrent common bile duct stone on contrast-enhanced computed tomography ( Fig. 1 ). A side-viewing duodenoscope could not be passed into the duodenum owing to sharp bowel angulation resulting from adhesions. Therefore, a passive-bending colonoscope (PCF-H290ZI; Olympus Corp., Tokyo, Japan) was maneuvered into the duodenum. After the duodenum was reached, a double-guidewire was advanced through to the jejunum ( Fig. 2 a ). The colonoscope was exchanged for a side-viewing duodenoscope (TJF-Q290V; Olympus Corp) with the wire left in place (over-the-wire technique). Under fluoroscopic and endoscopic visualization, a duodenoscope was carefully pushed and passed into the duodenum ( Fig. 2 b ), and the double-guidewire was removed. Successful biliary cannulation was performed using the pancreatic guidewire placement method ( Fig. 2 c ). After a prophylactic pancreatic stent placement, a sphincterotomy with stone removal was successfully achieved ( Fig. 2 d ). Six days after the ERCP, the pancreatic stent was removed ( Video 1 ).

**Fig. 1:**
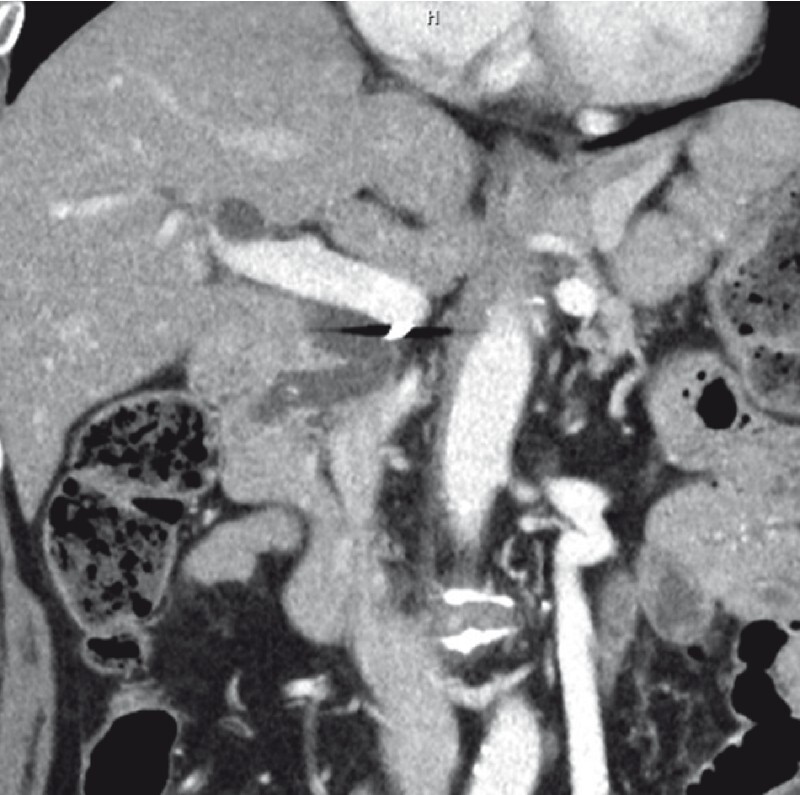
Computed tomography (CT) revealed a stone in the tortuous bile duct.

**Fig. 2 a:**
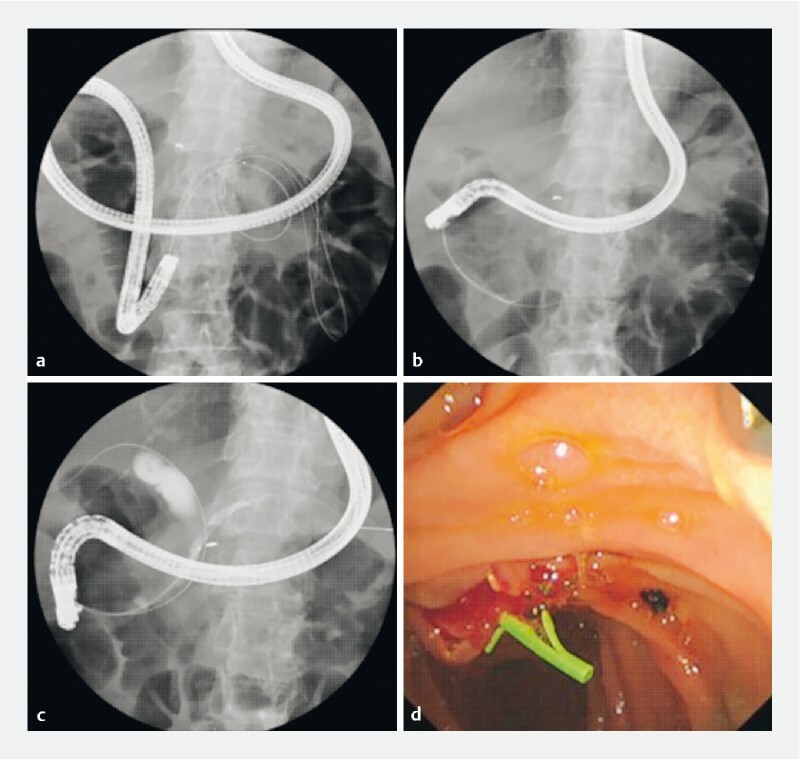
A passive bending colonoscope was passed into the duodenum, and a double guidewire was advanced through the jejunum. **b** A duodenoscope was passed into the duodenum. **c** Biliary cannulation was achieved using a pancreatic guidewire placement technique. **d** Stone removal was performed after endoscopic sphincterotomy.

**Video 1** Endoscopic removal by over-the-wire technique for common bile duct stone after total gastrectomy with jejunal interposition.


This case demonstrates an over-the-wire technique that is effective in ERCP after total gastrectomy with jejunal interposition. By leaving the wire in place, the duodenoscope can be safely advanced under endoscopic and fluoroscopic guidance.

Endoscopy_UCTN_Code_TTT_1AR_2AB

## Notes

Endoscopy E-Videos

https://eref.thieme.de/e-videos
*E-Videos* is an open access online section of the journal *Endoscopy* , reporting on interesting cases and new techniques in gastroenterological endoscopy. All papers include a high-quality video and are published with a Creative Commons CC-BY license. Endoscopy E-Videos qualify for HINARI discounts and waivers and eligibility is automatically checked during the submission process. We grant 100% waivers to articles whose corresponding authors are based in Group A countries and 50% waivers to those who are based in Group B countries as classified by Research4Life (see: https://www.research4life.org/access/eligibility/ ). This section has its own submission website at https://mc.manuscriptcentral.com/e-videos
